# A case of Schloffer tumor with rapid growth and FDG-PET positivity at the port site of laparoscopic sigmoidectomy for colon cancer

**DOI:** 10.1186/s40792-019-0677-7

**Published:** 2019-07-23

**Authors:** Eisuke Asano, Yumi Furuichi, Kensuke Kumamoto, Jun Uemura, Takayoshi Kishino, Hisashi Usuki, Keiichi Okano, Yasuyuki Suzuki

**Affiliations:** Department of Gastroenterological Surgery, Faculty of Medicine, Kagawa University, 1750-1 Ikenobe, Miki-cho, Kita-Gun, Kagawa, 761-0793 Japan

**Keywords:** Schloffer tumor, Laparoscopic sigmoidectomy, Port site recurrence

## Abstract

**Background:**

Schloffer tumor is a foreign body granuloma in the abdominal subcutaneous layer that develops due to a foreign body such as suture from several months to years postoperatively. Herein, we report a case of a rapidly growing Schloffer tumor with F-18 fluorodeoxyglucose (FDG) positron emission tomography (PET) positivity at the port site of laparoscopic sigmoidectomy for colon cancer.

**Case presentation:**

An 85-year-old man, who underwent laparoscopic sigmoidectomy for stage IIIa sigmoid colon cancer 10 months ago, was referred to our hospital with complaints of a growing mass in the abdominal wall. The tumor was palpable at the right-sided abdominal wall corresponding to the port site of laparoscopic sigmoidectomy. The tumor rapidly grew for 2 months. Computed tomography showed a ring-enhanced mass at the right-sided abdominal wall. PET examination revealed high accumulation of FDG in the tumor. Tumor resection was performed due to suspected port site recurrence. The pathological diagnosis was inflammatory granuloma, so-called Schloffer tumor.

**Conclusion:**

In the era of laparoscopic surgery, Schloffer tumor may be one of the differential diagnoses for rapidly growing tumor with FDG-PET positivity at the port site in postoperative patients with advanced colorectal cancer.

## Background

Schloffer tumor has been originally reported as inflammatory tumors in five cases after hernia surgery in 1909 [1] and is defined as a foreign body granuloma that usually occurs due to the stimulation of a foreign body used for surgery at the abdominal scar from several months to years after an abdominal surgery. Recently, laparoscopic surgery has become popular for gastroenterological cancers. Although the frequency of port site recurrence is reportedly very low [2], a growing mass at the port site of laparoscopic surgery must be suspected with the recurrence of primary gastroenterological cancer. Previous studies [3–5] demonstrated that Schloffer tumor showed F-18 fluorodeoxyglucose (FDG) positron emission tomography (PET) positivity after the surgery for colorectal cancer. Therefore, distinguishing metastatic from Schloffer tumor is difficult when a growing mass at the port site of laparoscopic surgery is observed. In this report, we showed a case of a rapidly growing Schloffer tumor with FDG-PET positivity at the port site of laparoscopic sigmoidectomy for colon cancer.

## Case presentation

An 85-year-old man complained of a growing mass at the port site of laparoscopic sigmoidectomy in the abdominal subcutaneous layer from 1 month ago during his regular clinic visits. He had no fever and chills or any history of pain. He underwent laparoscopic sigmoidectomy for sigmoid colon cancer with pStage IIIa (S, type 2, 30 × 20 mm, pT3pN1) 10 months ago in our institution. He also had hepatocellular carcinoma (cT2N0M0, cStage II) and underwent transcatheter arterial chemoembolization. In routine laboratory tests, both white blood cell (WBC) count and C-reactive protein (CRP) values were within normal limits. Tumor marker values, including CEA, CA19-9, AFP, and PIVKA-2, were also within normal limits. A solid mass of approximately 2 cm in size was palpable without skin color changes at the right-sided port site. The tumor mobility was relatively good and its surface appeared to be irregular.

**Fig. 1:**
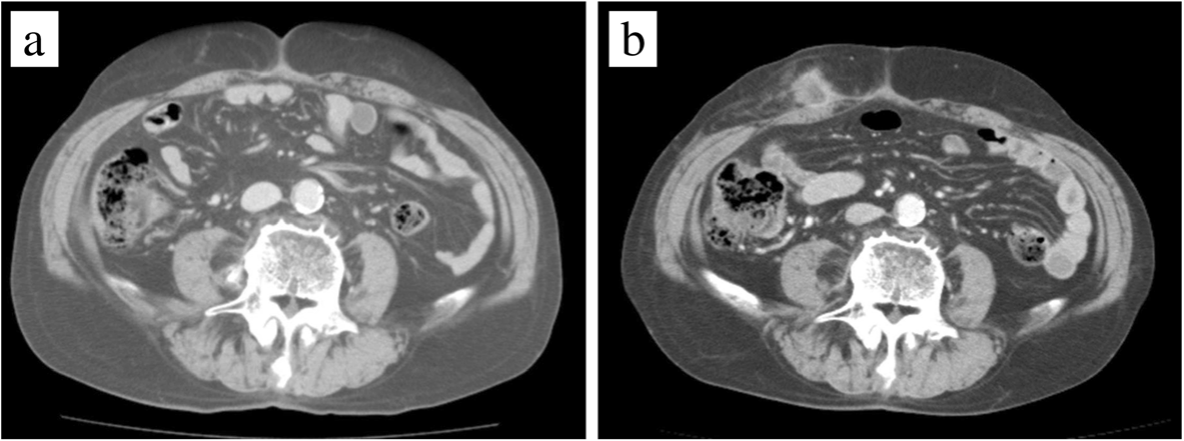
An abdominal contrast-enhanced computed tomography (CT). **a** Two months before the initial diagnosis of Schloffer tumor. **b** Pre-operative CT scan showed a ring-enhanced mass at the ventral side of the right abdominal wall

**Fig. 2:**
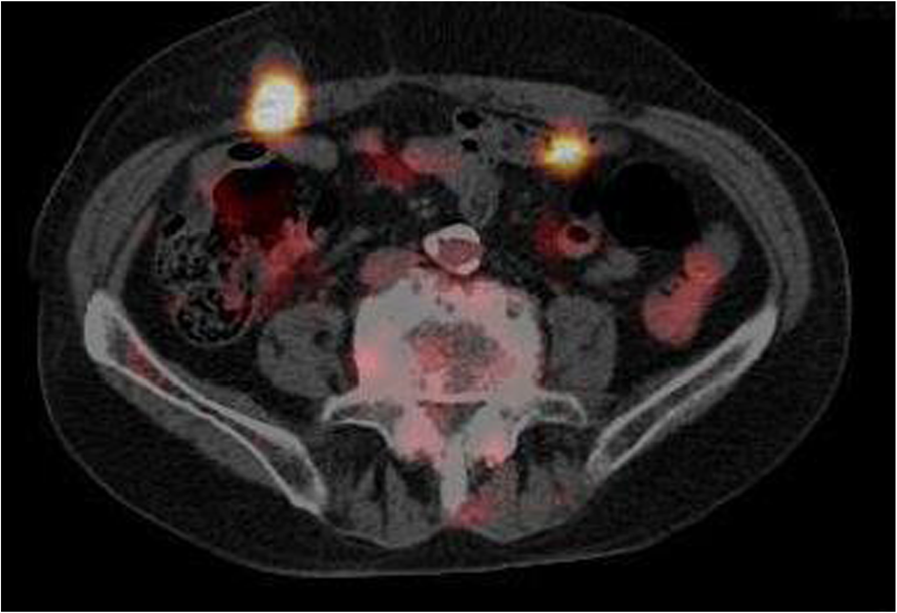
Pre-operative PET-CT showed high accumulation of FDG (SUV_max_ 14.6) at the ventral side of the right abdominal wall

**Fig. 3:**
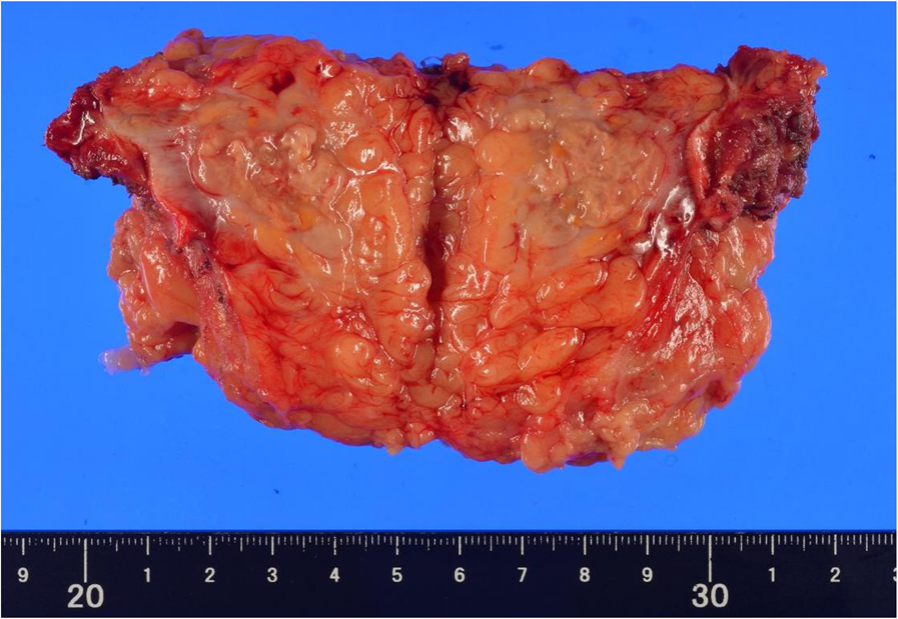
Resected specimen. The center of the tumor showed necrotic tissue

**Fig. 4:**
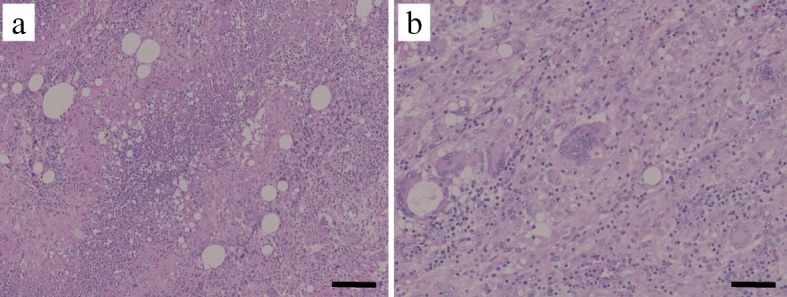
Representative histological photographs of resected specimen (hematoxylin and eosin staining). **a** Main area of the tumor revealed the inflammatory cell infiltration (× 50, bar; 500 μm). **b** Langhans giant cells (× 400, bar; 50 μm)

The tumor appears to be rapidly growing because it was not yet detected in the computerized tomography (CT) performed 2 months ago (Fig. 1a), during the 6-month postoperative follow-up for colon cancer. Ultrasonography of the subcutaneous mass showed a 2-cm hypoechoic lesion in contact with the abdominal wall. Enhanced abdominal CT showed a ring-enhanced mass at the right side of the abdominal subcutaneous layer (Fig. 1b). Other metastatic lesions or recurrence of hepatocellular carcinoma lesion were not observed. In PET examination, high accumulation of FDG was observed in the tumor with SUV_max_ of 14.6 (Fig. 2). Based on the findings of clinical imaging and clinical course, port site recurrence was suspected after a laparoscopic sigmoidectomy for advanced colon cancer. He underwent radical tumor resection. The resected specimen was an elastic hard and solid nodule (Fig. 3). No foreign body was found in the mass. Pathological findings revealed many Langhans giant cells and monocytes in the background of inflammatory granuloma (Fig. 4a, b). Consequently, the tumor was diagnosed as Schloffer tumor.

## Discussion

The Schloffer tumor is difficult to diagnose preoperatively when a rapidly growing tumor was found at the operative scar after cancer surgery. In this case, the tumor was rapidly growing at the port site scar in 2 months and demonstrated FDG-PET positivity. The possibility for port site recurrence cannot be ruled out as advanced colon cancer, which was curatively resected 10 months ago. Therefore, tumor resection was performed without biopsy, which showed it was a foreign body granuloma based on pathological examination.

Schloffer tumor is a foreign body granuloma with chronic inflammation that usually occurs by the stimulation of a foreign body used for surgery at an operative scar of the abdominal wall. The occurrence rate of granuloma postoperatively with non-absorbable suture is higher than that with absorbable suture [6]. Absorbable intra-abdominal suture is more suitable than silk in reducing the surgical site infection, especially in colorectal surgery [7]. Recently, laparoscopic surgery has become popular because it can be performed with small incision and less suture compared to laparotomy, suggesting that external factors forming a foreign body granuloma have been decreasing with time, but patient risks still remain. In our case, Schloffer tumor was located below the 5 mm-wound at the right side of the navel that has only skin suture by absorbable monofilament polydioxanone. The thread was considered to be absorbed and disappeared because it had been present 10 months postoperatively. Therefore, no foreign body could be macroscopically found in the tumor. The patient had no allergic characteristics, suggesting that absorbable suture may form a foreign body granuloma although the suture has disappeared.

In this case, high accumulation of ^18^F-FDG was observed in the tumor without increased WBC count and CRP value. Preoperatively, port site recurrence was suspected due to postoperative colon cancer with advanced stage. Lim et al. reported that the frequency of port site metastasis in colorectal cancer was 0.18% in 2011 [8]. Patients with advanced stage of colon cancer are at higher risk of port site recurrence [9]. The PET examination is usually performed for cancer recurrence screening. Previous reports demonstrated that PET examination was useful for the early diagnosis of port site recurrence of colorectal cancer [10, 11]. On the contrary, PET examination is limited because of false-positive findings for inflammation, such as suture granuloma [12]. Although the frequency of port site recurrence is very low, distinguishing malignant from inflammatory tumors may be difficult prior to surgical treatment. We have to consider that Schloffer tumor shows FDG-PET positivity.

Based on the literature search of PubMed (using keywords, “Schloffer tumor” and “port site recurrence”), no cases have been detected. We then found 3 Japanese studies [3, 4, 13] on Schloffer tumor with postoperative colorectal cancer. The clinicopathological findings of the reported cases, including our case, are summarized in Table 1. Among these three reports, one case (no. 3 in Table 1) was similar to our case in terms of clinical features. Majority of tumors occurs within 1 year. Tumors in all cases were FDG-PET positive without an increase in WBC count, CRP value, and tumor marker values. No foreign body was found in all tumors. The absorbable suture was used for all cases, leading to suggest that absorbable suture may result in the formation of foreign body granuloma. The biopsy was performed in one case (no. 3). However, they could not deny the possibility of malignant potential in a growing tumor. In our case, biopsy was not performed due to his clinical course. However, the tumor may be removed under local anesthesia if the pathological finding was negative for malignant cells by biopsy.

**Table 1:** Previous reports of Schloffer tumor at abdominal operative scar in patients who underwent colorectal surgery

	Author	Reported year	Age	Gender	Primary cancer	Stage	Tumor location	Interval to occurrence (months)	Tumor size (mm)	Biopsy	Suture	FDG-PET	WBC CRP	CEA CA19-9
1	Shibata	2006	73	F	Sigmoid cancer	II	Lower abdominal wall	101	22 × 20	n.d.	Not detected	Positive	WNL	WNL
2	Maeda	2007	47	M	Rectal cancer	IIIa	Lower abdominal midline surgical wound	12	20	n.d.	Not detected	Positive	WNL	WNL
3	Tsukamoto	2014	83	M	Rectal cancer	IIIa	Port site of navel	10	30	Negative	Not detected	Positive	WNL	WNL
4	Our case	2019	85	M	Sigmoid cancer	IIIa	Port site of right-sided wall	10	20	n.d.	Not detected	Positive	WNL	WNL

*n.d.* not done, *WNL* within normal limits

## Conclusion

In the era of laparoscopic surgery, Schloffer tumor may be one of the differential diagnoses for a rapidly growing tumor with FDG-PET positivity at the port site in postoperative patients with advanced colorectal cancer even if an absorbable suture is used.

## Data Availability

The authors declare that all the data in this article are available within the article.

## Abbreviations

CRP: C-reactive protein
CT: Computed tomography
FDG: F-18 fluorodeoxyglucose
PET: Positron emission tomography
WBC: White blood cell

## References

[1] Schloffer H (1909). Ueber chronisch entz ndliche Bauchdeckentumoren nach Hernienoperationen. Arch Klin Chir..

[2] Green BL, Marshall HC, Collinson F, Quirke P, Guillou P, Jayne DG (2013). Long-term follow-up of the Medical Research Council CLASICC trial of conventional versus laparoscopically assisted resection in colorectal cancer. Br J Surg..

[3] Shibata T, Katsuramaki T, Mizuguchi T, Kaji S, Hirata K (2006). A case of Schloffer tumor with false-positive results in PET screening. Nihon Rinsyo Geka Gakkai Zasshi.

[4] Maeda K, Adachi A, Hashimoto N, Takano N, Uchiyama T (2007). A case of a Schloffer tumor and a Braun tumor which were difficult to differentiate from metastatic tumors due to FDG-PET positive. Nihon Rinsyo Geka Gakkai Zasshi.

[5] Ohta H, Mochizuki K, Tsukayama S, Kawamura Y, Sonoda H, Shimizu T (2014). Two cases of Schloffer tumor, initially suspected to be recurrence demonstrating PET false positive, after surgery for colon cancer. J Jpn Soc Coloproctol.

[6] Iwase K, Higaki J, Tanaka Y, Kondoh H, Yoshikawa M, Kamiike W (1999). Running closure of clean and contaminated abdominal wounds using a synthetic monofilament absorbable looped suture. Surg Today..

[7] Watanabe A, Kakeji Y, Taketomi A, Emi Y, Kohnoe S, Maehara Y (2009). Silk versus polyglactin (Vicryl®) in intra-abdominal suture: a prospective comparative study. J.Jpn.Soc.Surg.Infect..

[8] Lim SW, Cho SH, Oh BR, Huh JW, Kim YJ, Kim HR (2011). Port-site recurrence after laparoscopy-assisted low anterior resection: the sign of peritoneal dissemination. Int J Colorectal Dis..

[9] Wexner SD, Cohen SM (1995). Port site metastasis after laparoscopic colorectal surgery for cure of malignancy. Br. J Surg..

[10] Funahashi K, Ushigome M, Kaneko H (2011). A role of 18F-fluorodeoxyglucose positron emission/computed tomography in a strategy for abdominal wall metastasis of colorectal mucinous adenocarcinoma developed after laparoscopic surgery. World J Surg Oncol..

[11] Kosugi C, Ono M, Saito N, Sugito M, Ito M, Murakami K (2005). Port site recurrence diagnosed by positron emission tomography after laparoscopic surgery for colon cancer. Hepatogastroenterology.

[12] Takeshita N, Tohma T, Miyauchi H, Suzuki K, Nishimori T, Ohira G (2015). Suture granuloma with false-positive findings on FDG-PET/CT resected via laparoscopic surgery. Int Surg.

[13] Tsukamoto Y, Nakao T (2014). A case of Schloffer tumor at a port site after resection for rectal cancer. Nihon Rinsyo Geka Gakkai Zasshi.

